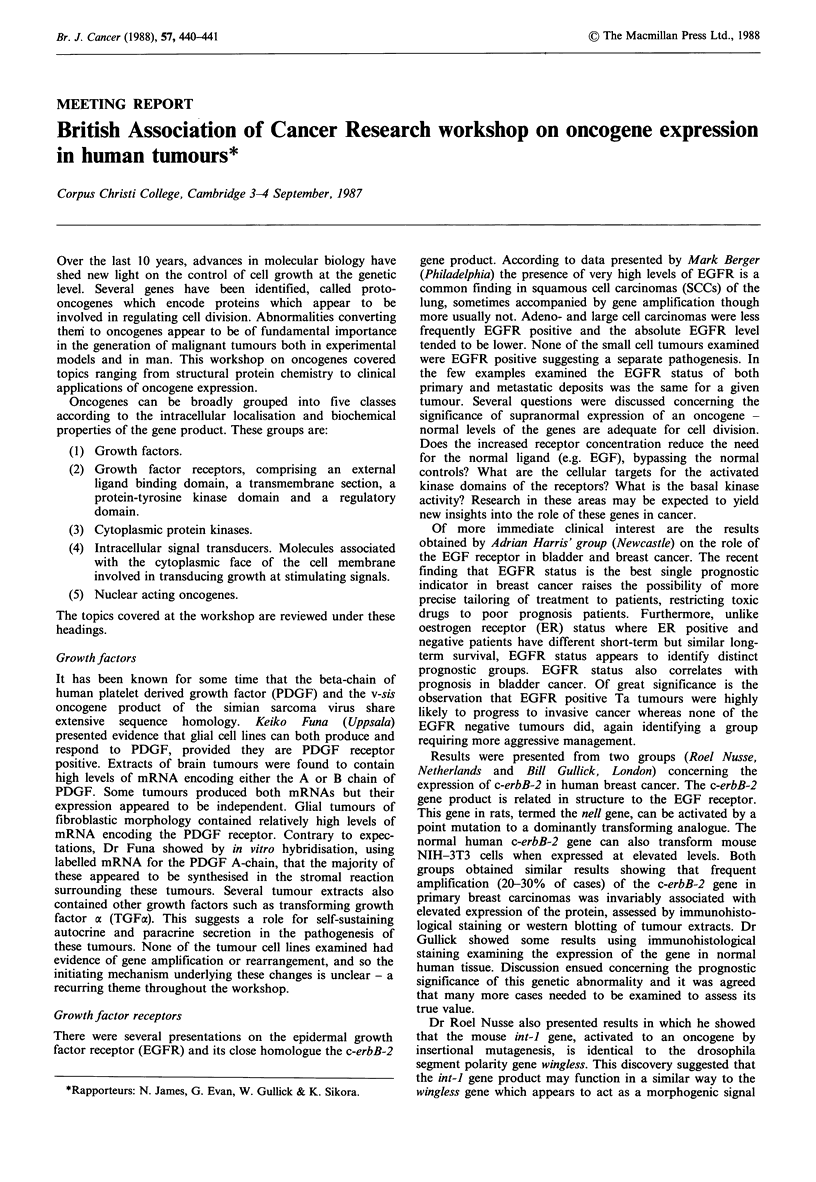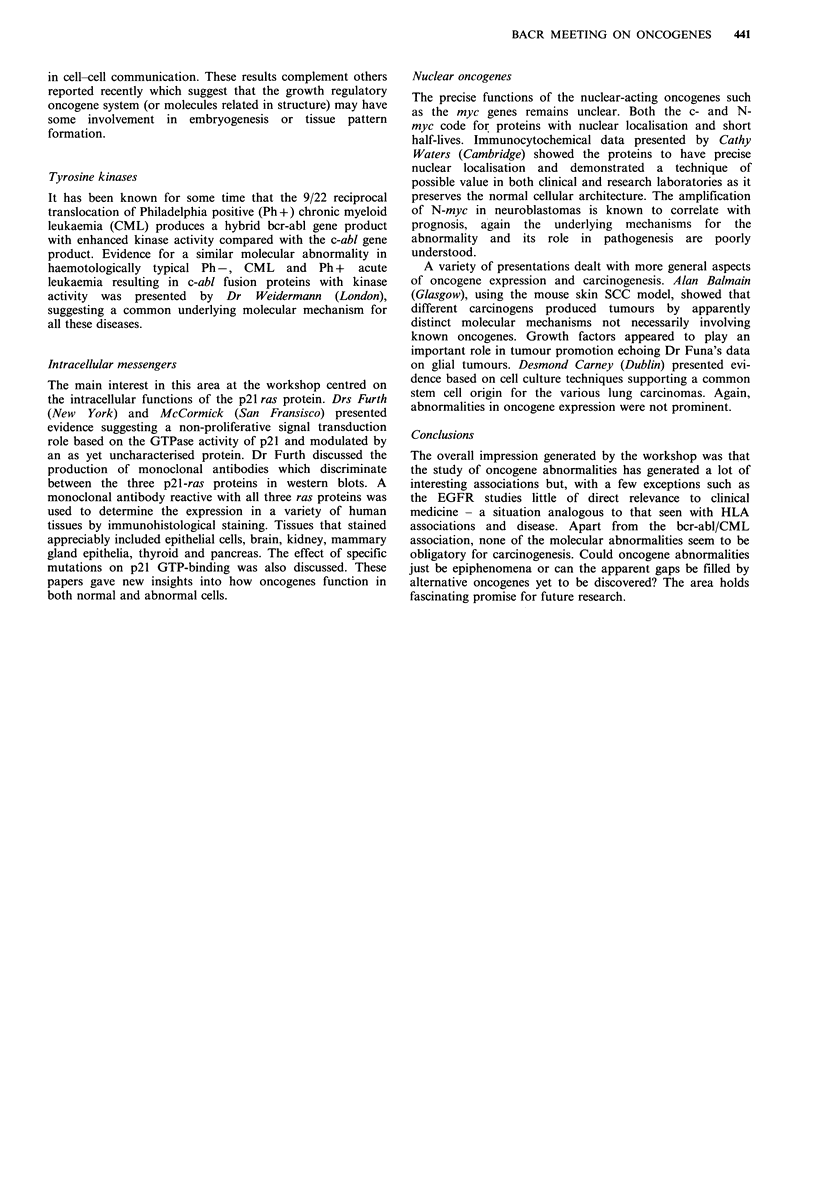# BACR workshop on oncogene expression in human tumours*

**Published:** 1988-04

**Authors:** 


*Br. J. Cancer* (1988), **57**, 440–441 © The Macmillan Press Ltd., 1988

MEETING REPORT

### British Association of Cancer Research workshop on oncogene expression in human tumours*

*Corpus Christi College, Cambridge 3–4 September, 1987*

Over the last 10 years, advances in molecular biology have shed new light on the control of cell growth at the genetic level. Several genes have been identified, called proto-oncogenes which encode proteins which appear to be involved in regulating cell division. Abnormalities converting them to oncogenes appear to be of fundamental importance in the generation of malignant tumours both in experimental models and in man. This workshop on oncogenes covered topics ranging from structural protein chemistry to clinical applications of oncogene expression.

Oncogenes can be broadly grouped into five classes according to the intracellular localisation and biochemical properties of the gene product. These groups are:

(1) Growth factors.
(2) Growth factor receptors, comprising an external ligand binding domain, a transmembrane section, a protein-tyrosine kinase domain and a regulatory domain.
(3) Cytoplasmic protein kinases.
(4) Intracellular signal transducers. Molecules associated with the cytoplasmic face of the cell membrane involved in transducing growth at stimulating signals.
(5) Nuclear acting oncogenes.

The topics covered at the workshop are reviewed under these headings.

### *Growth factors*

It has been known for some time that the beta-chain of human platelet derived growth factor (PDGF) and the v-*sis* oncogene product of the simian sarcoma virus share extensive sequence homology. *Keiko Funa* (*Uppsala*) presented evidence that glial cell lines can both produce and respond to PDGF, provided they are PDGF receptor positive. Extracts of brain tumours were found to contain high levels of mRNA encoding either the A or B chain of PDGF. Some tumours produced both mRNAs but their expression appeared to be independent. Glial tumours of fibroblastic morphology contained relatively high levels of mRNA encoding the PDGF receptor. Contrary to expectations, Dr Funa showed by *in vitro* hybridisation, using labelled mRNA for the PDGF A-chain, that the majority of these appeared to be synthesised in the stromal reaction surrounding these tumours. Several tumour extracts also contained other growth factors such as transforming growth factor α (TGFα). This suggests a role for self-sustaining autocrine and paracrine secretion in the pathogenesis of these tumours. None of the tumour cell lines examined had evidence of gene amplification or rearrangement, and so the initiating mechanism underlying these changes is unclear – a recurring theme throughout the workshop.

### *Growth factor receptors*

There were several presentations on the epidermal growth factor receptor (EGFR) and its close homologue the c-*erbB-2* gene product. According to data presented by *Mark Berger* (*Philadelphia*) the presence of very high levels of EGFR is a common finding in squamous cell carcinomas (SCCs) of the lung, sometimes accompanied by gene amplification though more usually not. Adeno- and large cell carcinomas were less frequently EGFR positive and the absolute EGFR level tended to be lower. None of the small cell tumours examined were EGFR positive suggesting a separate pathogenesis. In the few examples examined the EGFR status of both primary and metastatic deposits was the same for a given tumour. Several questions were discussed concerning the significance of supranormal expression of an oncogene – normal levels of the genes are adequate for cell division. Does the increased receptor concentration reduce the need for the normal ligand (e.g. EGF), bypassing the normal controls? What are the cellular targets for the activated kinase domains of the receptors? What is the basal kinase activity? Research in these areas may be expected to yield new insights into the role of these genes in cancer.

Of more immediate clinical interest are the results obtained by *Adrian Harris' group* (*Newcastle*) on the role of the EGF receptor in bladder and breast cancer. The recent finding that EGFR status is the best single prognostic indicator in breast cancer raises the possibility of more precise tailoring of treatment to patients, restricting toxic drugs to poor prognosis patients. Furthermore, unlike oestrogen receptor (ER) status where ER positive and negative patients have different short-term but similar long-term survival, EGFR status appears to identify distinct prognostic groups. EGFR status also correlates with prognosis in bladder cancer. Of great significance is the observation that EGFR positive Ta tumours were highly likely to progress to invasive cancer whereas none of the EGFR negative tumours did, again identifying a group requiring more aggressive management.

Results were presented from two groups (*Roel Nusse, Netherlands* and *Bill Gullick, London*) concerning the expression of c-*erbB-2* in human breast cancer. The c-*erbB-2* gene product is related in structure to the EGF receptor. This gene in rats, termed the *nell* gene, can be activated by a point mutation to a dominantly transforming analogue. The normal human c-*erbB-2* gene can also transform mouse NIH-3T3 cells when expressed at elevated levels. Both groups obtained similar results showing that frequent amplification (20–30% of cases) of the c-*erbB-2* gene in primary breast carcinomas was invariably associated with elevated expression of the protein, assessed by immunohistological staining or western blotting of tumour extracts. Dr Gullick showed some results using immunohistological staining examining the expression of the gene in normal human tissue. Discussion ensued concerning the prognostic significance of this genetic abnormality and it was agreed that many more cases needed to be examined to assess its true value.

Dr Roel Nusse also presented results in which he showed that the mouse *int-1* gene, activated to an oncogene by insertional mutagenesis, is identical to the drosophila segment polarity gene *wingless*. This discovery suggested that the *int-1* gene product may function in a similar way to the *wingless* gene which appears to act as a morphogenic signal

*Rapporteurs: N. James, G. Evan, W. Gullick & K. Sikora.

in cell–cell communication. These results complement others reported recently which suggest that the growth regulatory oncogene system (or molecules related in structure) may have some involvement in embryogenesis or tissue pattern formation.

### *Tyrosine kinases*

It has been known for some time that the 9/22 reciprocal translocation of Philadelphia positive (Ph+) chronic myeloid leukaemia (CML) produces a hybrid bcr-abl gene product with enhanced kinase activity compared with the c-*abl* gene product. Evidence for a similar molecular abnormality in haemotologically typical Ph−, CML and Ph+ acute leukaemia resulting in c-*abl* fusion proteins with kinase activity was presented by *Dr Weidermann* (*London*), suggesting a common underlying molecular mechanism for all these diseases.

### *Intracellular messengers*

The main interest in this area at the workshop centred on the intracellular functions of the p21 *ras* protein. *Drs Furth* (*New York*) and *McCormick* (*San Fransisco*) presented evidence suggesting a non-proliferative signal transduction role based on the GTPase activity of p21 and modulated by an as yet uncharacterised protein. Dr Furth discussed the production of monoclonal antibodies which discriminate between the three p21-*ras* proteins in western blots. A monoclonal antibody reactive with all three *ras* proteins was used to determine the expression in a variety of human tissues by immunohistological staining. Tissues that stained appreciably included epithelial cells, brain, kidney, mammary gland epithelia, thyroid and pancreas. The effect of specific mutations on p21 GTP-binding was also discussed. These papers gave new insights into how oncogenes function in both normal and abnormal cells.

### *Nuclear oncogenes*

The precise functions of the nuclear-acting oncogenes such as the *myc* genes remains unclear. Both the c- and N-*myc* code for proteins with nuclear localisation and short half-lives. Immunocytochemical data presented by *Cathy Waters* (*Cambridge*) showed the proteins to have precise nuclear localisation and demonstrated a technique of possible value in both clinical and research laboratories as it preserves the normal cellular architecture. The amplification of N-*myc* in neuroblastomas is known to correlate with prognosis, again the underlying mechanisms for the abnormality and its role in pathogenesis are poorly understood.

A variety of presentations dealt with more general aspects of oncogene expression and carcinogenesis. *Alan Balmain* (*Glasgow*), using the mouse skin SCC model, showed that different carcinogens produced tumours by apparently distinct molecular mechanisms not necessarily involving known oncogenes. Growth factors appeared to play an important role in tumour promotion echoing Dr Funa's data on glial tumours. *Desmond Carney* (*Dublin*) presented evidence based on cell culture techniques supporting a common stem cell origin for the various lung carcinomas. Again, abnormalities in oncogene expression were not prominent.

### *Conclusions*

The overall impression generated by the workshop was that the study of oncogene abnormalities has generated a lot of interesting associations but, with a few exceptions such as the EGFR studies little of direct relevance to clinical medicine – a situation analogous to that seen with HLA associations and disease. Apart from the bcr-abl/CML association, none of the molecular abnormalities seem to be obligatory for carcinogenesis. Could oncogene abnormalities just be epiphenomena or can the apparent gaps be filled by alternative oncogenes yet to be discovered? The area holds fascinating promise for future research.